# High-performance n-type black phosphorus transistors with type control via thickness and contact-metal engineering

**DOI:** 10.1038/ncomms8809

**Published:** 2015-07-30

**Authors:** David J. Perello, Sang Hoon Chae, Seunghyun Song, Young Hee Lee

**Affiliations:** 1 IBS Center for Integrated Nanostructure Physics, Institute for Basic Science, Sungkyunkwan University, Suwon 440-746, Korea; 2 Department of Energy Science, Sungkyunkwan University, Suwon 440-746, Korea; 3 Department of Physics, Sungkyunkwan University, Suwon 440-746, Korea

## Abstract

Recent work has demonstrated excellent p-type field-effect switching in exfoliated black phosphorus, but type control has remained elusive. Here, we report unipolar n-type black phosphorus transistors with switching polarity control via contact-metal engineering and flake thickness, combined with oxygen and moisture-free fabrication. With aluminium contacts to black phosphorus, a unipolar to ambipolar transition occurs as flake thickness increases from 3 to 13 nm. The 13-nm aluminium-contacted flake displays graphene-like symmetric hole and electron mobilities up to 950 cm^2^ V^−1^ s^−1^ at 300 K, while a 3 nm flake displays unipolar n-type switching with on/off ratios greater than 10^5^ (10^7^) and electron mobility of 275 (630) cm^2^ V^−1^ s^−1^ at 300 K (80 K). For palladium contacts, p-type behaviour dominates in thick flakes, while 2.5–7 nm flakes have symmetric ambipolar transport. These results demonstrate a leap in n-type performance and exemplify the logical switching capabilities of black phosphorus.

## Abstract (web summary)

Black phosphorus p-type field-effect switching was previously demonstrated, but type control has proven difficult. Here, the authors create n-type black phosphorus Schottky field-effect transistors in which the polarity is controlled via contact-metal engineering and changing the flake thickness.

## Graphical abstract

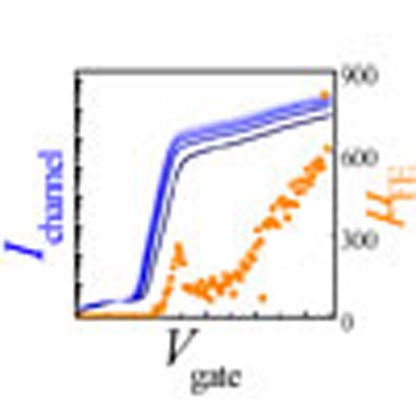

Ultrathin black phosphorus (BP), colloquially referred to as phosphorene, is a layered allotrope of phosphorus with a highly anisotropic and thickness-dependent band structure^1^,^2^,^3^,^4^,^5^. In contrast to semiconducting transition metal dichalcogenides with typically large bandgap and small mobility, or zero gap and high-mobility monolayer graphene, BP has both high mobility^1^ (in bulk) and a bandgap ranging from 0.3 to 0.39 eV in bulk to∼1.5–2 eV for monolayer^3^,^4^,^5^,^6^,^7^. Currently, thin layers can be accessed only from exfoliation of bulk crystalline BP samples, as no chemical vapor deposition (CVD) or epitaxial synthesis method exists. Despite this limitation, initial reports for exfoliated flakes between 2 and 20 nm thick have demonstrated that the material can be used as a high-performance p-type conducting channel with room temperature hole mobilities (*μ*_FE_) of 10^2^–10^3^ cm^2^ V^−1^ s^−1^(refs 8, 9, 10, 11, 12, 13, 14). Intrinsically however, theory predicts that pristine BP displays no dominant preference to carrier type. Thus far, only p-type unipolar and p-type dominant ambipolar transport has been observed due to suppressed electron transport, resulting from a combination of oxygen and moisture exposure^15^,^16^,^17^,^18^,^19^,^20^,^21^. To date, no n-type transistor operation or clear mechanism of type control has been reported^8^.

In the following, we demonstrate that BP can serve as a high-performance n-type, ambipolar, or p-type transistor channel dependent on both flake thickness and contact metal. Theoretical layer dependence of the valence and conduction band edges^3^ (*E*_C_ and *E*_V_, respectively) is exploited to fabricate high-performance n-type FETs with electron mobilities of 10^2^–10^3^ cm^2^ V^−1^ s^−1^ at 300 K using Al as a Schottky-type contact. With Al contacts, ultrathin 3-nm flakes show the most promise for use in logic circuits, with a combined *I*_on_/*I*_off_>10^5^ (10^7^) and electron mobility of 275 (630) cm^2^ V^−1^ s^−1^ at 300 K (80 K). Al–BP Schottky-field-effect transistors (Schottky FETs) are contrasted with Pd–BP samples with lower contact resistance. With Pd contacts, FETs are channel resistance-dominant devices and more intrinsic properties of the flakes can be extracted. These devices display a p-type to symmetric ambipolar transition when the thickness of the BP is reduced. The influence of dielectric coating, orientation and dual gating on carrier type and device performance is further investigated.

## Results

### Al-contacted n-type FETs

**Figure 1:**
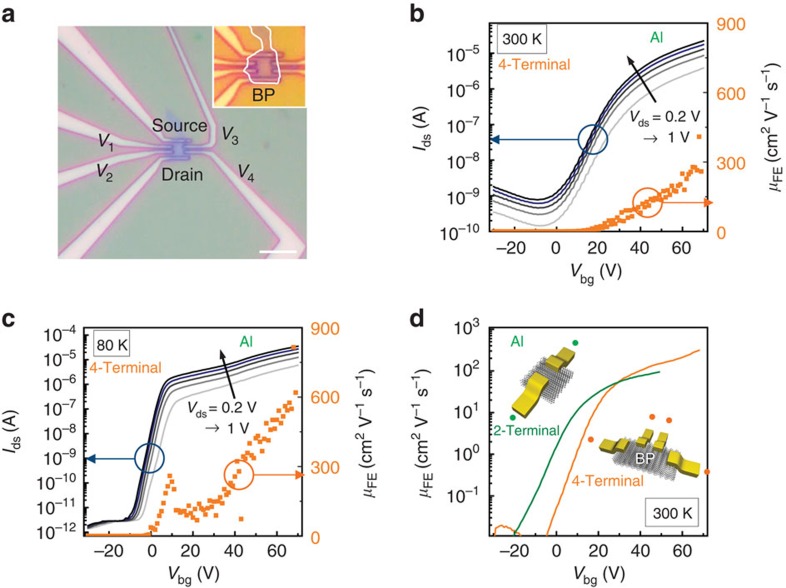
High-performance n-type transistors with Al contacts. (**a**) Optical image of the device for four-probe measurements. Scale bar is 6 μm. (**b**) *I*_ds_ showing n-type operation with *I*_on_/*I*_off_∼10^5^ at 300 K. The n-type 4-terminal-calculated *μ*_FE_ at 300 K is 275 cm^2^ V^−1^ s^−1^. (**c**) *I*_ds_ showing n-type operation with *I*_on_/*I*_off_>10^7^ at 80 K. The n-type 4-terminal mobility is *μ*_FE_∼630 cm^2^ V^−1^ s^−1^ at 80 K with no sign of saturation. (**d**) Comparison of 2-terminal and 4-terminal field-effect mobilities. Contact resistance is the source of this difference.

Transport measurements of BP-FETs are performed in both 4-terminal and 2-terminal configurations to extract contact and channel properties of the BP-FETs. Figure 1a shows a 6-terminal hall bar fabricated on a 3-nm thick flake with Al(10 nm)/Cr(3 nm)/Au(20 nm) contact-metal stack. Al was chosen as the contact material due to its low work function (*Φ*_M_∼4.0–4.3 eV) and lengthy history as a low-resistance n-type contact to semiconductors like silicon^22^. In contrast to heavily doped silicon, thin intrinsic BP is highly conductive without a fixed majority carrier. Therefore, with Al contacts, *I*_ds_–*V*_bg_ (d,s,bg subscripts denote the drain, source, and back gate, respectfully) measurements display clear Schottky-FET behaviour. This is shown at 300 K in Fig. 1b, where unipolar behaviour and an extremely large but unsaturated (4-terminal) electron carrier mobility *μ*_FE_=275 cm^2^ V^−1^ s^−1^ at *V*_bg_=70 V is observed. Hole transport is suppressed such that *I*_on_/*I*_off_∼10^5^ at 300 K, which is further enhanced to 10^7^ at 80 K, as shown in Fig. 1c. Notably, peak electron mobility (4-terminal) is enhanced to 630 cm^2^ V^−1^ s^−1^ at 80 K (*V*_bg_=70 V), while band-edge mobility at *V*_bg_=9 V increases from 1 to 290 cm^2^ V^−1^ s^−1^. Hole mobilities show no improvement with decreased temperature. The subthreshold swing (SS), which is described by SS=ln(10)*kT*(1+*C*_BP_/
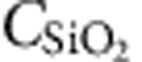
), shows a temperature-dependent reduction from 9 V per decade at 300 K to 2.5 V per decade at 80 K. Substituting into the above, the depletion capacitance of the 3.5 nm thick BP is *C*_BP_=1.62 μF cm^−2^.

To understand the influence of the contact resistance, we compare 4-terminal measurements with 2-terminal results on the same BP sample. At 50 V, two terminal peak mobility of 94 cm^2^ V^−1^ s^−1^ compares with the 4-terminal 150 cm^2^ V^−1^ s^−1^ electron mobility at 300 K. The 50% increase in the mobility results from the large contact resistance with Al. Improvements to n-type contact resistance while retaining suppressed hole transport will need to be addressed in further studies. All samples with Al contacts displayed n-type behaviour (other examples can be found in Supplementary Fig. 1).

**Figure 2:**
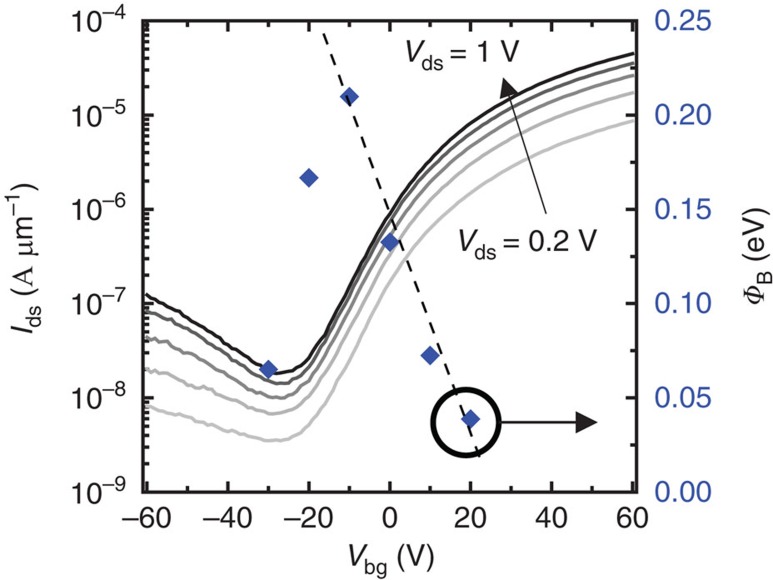
Al–BP Schottky barrier measurement. *I*_ds_–*V*_bg_ at 300 K for a 6-nm thick flake overlaid with the Schottky barrier height *Φ*_B_, which was extracted via activation energy measurements. The barrier at *V*_bg_=−30 V represents a tunneling barrier while *V*_bg_≥−10 V displays excellent fit with pure thermionic emission.

Confirmation of Schottky barrier origins of the n-type behaviour was then established by performing activation energy measurements (Supplementary Fig. 2) to extract equilibrium barrier heights (*Φ*_B_) at *V*_ds_=0 V for different gate potentials. Results for a 2-terminal n-type Al-contacted 6-nm thick BP flake are detailed in Fig. 2. For *V*_bg_≥−10 V, excellent fit with pure thermionic emission is achieved. Additionally, by linearly fitting the dependence of *Φ*_B_ on *V*_bg_, we find that ∂*Φ*_B_/∂*V*_bg_≈6 mV(*Φ*_B_)/*V*_bg_. This equates to *SS*=10 V per decade at 300 K, which is in agreement with the *I*_ds_–*V*_bg_ raw data in Fig. 2 and further demonstrates that the switching is contact dominant.

With *V*_bg_<−10 V, *Φ*_B_ displays weak or no *V*_ds_ dependence which results from an increased off-state channel resistance or a subsequent increase in field emission current as *V*_bg_ pushes the Fermi level into the valence band. Tunnelling is more likely in this case, since the flake is 6-nm thick and the *I*_ds_–*V*_bg_ sweep in Fig. 2 shows an exponential increase in current with *V*_ds_ at *V*_bg_<−30 V. For thinner devices, as in Fig. 1, tunnelling is prevented by a larger bandgap and thicker depletion region. The *V*_bg_ dependence found here mirrors the Schottky barrier analysis of p-type devices in two recent reports^23^,^24^, although barrier heights here are at equilibrium which is in contrast to the Arrhenius analysis^23^.

**Figure 3:**
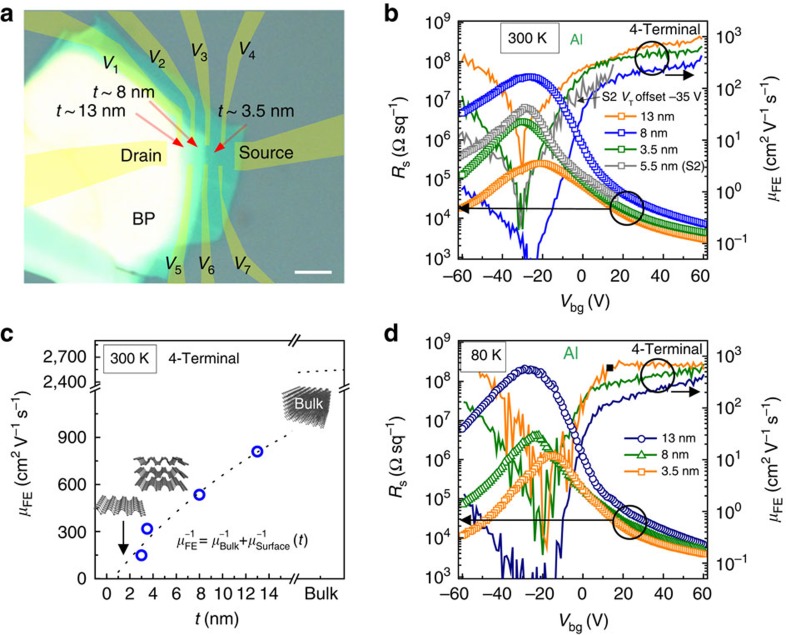
BP thickness dependences of Al-contacted devices. (**a**) Optical image of the BP flake (before processing) used for testing layer dependences of transport properties with Al contacts. Yellow shaded region shows locations of electrodes (Supplementary Fig. 3). Scale bar, 3 μm. (**b**) *R*_s_ (open points) and *μ*_FE_ (solid lines) from 4-terminal measurements at 300 K. (**c**) *μ*_FE_ at 300 K and *V*_bg_=50 V fit to Matthiessen's rule. From fitting, *μ*_Bulk_=2.6 × 10^3^ cm^2^ V^−1^ s^−1^ and estimated monolayer surface scattering is limited to 94 cm^2^ V^−1^ s^−1^. (**d**) *R*_s_ (open points) and *μ*_FE_ (solid lines) from 4-terminal measurements at 80 K. The n-type to ambipolar conversion is observed as the flake thickness increases. Mobility increases with increasing thickness, with n- and p-type *μ*_FE_∼9.5 × 10^2^ cm^2^ V^−1^ s^−1^ at 80 K for 13 nm thick region (*V*_1,2_ in **a**).

We next investigated the flake thickness dependence of transport with Al contacts on a separate elongated Hall bar geometry fabricated on the thickness-tapered edge of a larger flake (Fig. 3a). Optical image of the tapered flake with decorated electrode locations is shown in Fig. 3a.

BP thickness varies from 3.5 nm between probes *V*_34_ (*V*_67_), to 8 nm between probes *V*_23_ (*V*_56_), and up to 13 nm between *V*_12_. The thickness is not constant across the surface and thus an average estimate was used. Atomic force microscopy (AFM) is not ideal for determining the precise number of BP layers, but the exact height is not critical in obtaining general trends of transport, which can be determined by the relative differences in thickness on a single flake. 4-Terminal transport properties were measured at 300 K (Fig. 3b) and 80 K (Fig. 3d) to contrast sheet resistance (*R*_s_) and carrier mobilities for different thicknesses. These measurements show that 3.5-nm flakes retain the unipolar n-type transport similar to the device with a 3-nm flake in Fig. 1. As the flake thickness increases, electron mobility improves while a more noticeable increase in hole mobility occurs. For a 13-nm flake, transport is nearly ambipolar with an unsaturated and nearly symmetric electron and hole mobility of ∼950 cm^2^ V^−1^ s^−1^.

Due to the extreme instability of the BP, defining a precise mesa via an additional lithography and etching step was not performed. Time-to-measurement, air exposure and increased edge diffusion of oxygen and water molecules were deemed more problematic to the thickness-dependent relationship than mesa patterning^16^,^18^. Mobilities for the non-etched sample (Fig. 3a) were calculated based on an ideal assumption of no current spread across the flake due to the presence of Schottky contacts at all the voltage probe arms (*V*_1_–*V*_7_). This assumption can lead to overestimation of mobilities if the contact barriers are not large. In comparison, for samples with a defined mesa, a large positive threshold voltage (*V*_T_) shift typically occurs, preventing mobility saturation due to *V*_bg_ limitations. The 5.5-nm Al-contacted flake in Fig. 3b (gray) is one example and is shifted by −35 V to coincide with the non-etched flake. In comparing etched with non-etched samples, even without saturation, the peak mobility (250 cm^2^ V^−1^ s^−1^) of the etched sample fits within the thickness trend established for the non-etched flake in Fig. 3a. Therefore, the mobilities of the non-etched flake are not overtly overestimated. Mobility magnitude is also consistent with Fig. 1, which does not have such an unorthodox shape. Further discussion of mobility calculations can be found in Supplementary Fig. 3.

The thickness dependence of electron mobility is modelled via Matthiessen's rule in Fig. 3c: 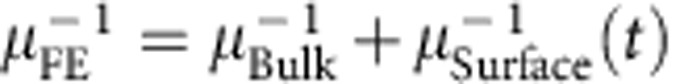, where *t* is the thickness of the flake measured from AFM measurement. We extract μ_Bulk_=2.6 × 10^3^ cm^2^ V^−1^ s^−1^ as *t* goes to infinity and μ_Mono_=94 cm^2^ V^−1^ s^−1^ at the monolayer limit. The monolayer estimate is extracted by assuming an AFM-measured thickness of *t*_Mono_=1.5 nm. A unit of 1.5 nm is chosen as an upper bound of monolayer thickness since the BP/SiO_2_ has ∼1 nm roughness in AFM scans, and the BP bulk layer spacing is 0.55 nm. The roughness and sample–tip interaction difference between SiO_2_ and BP can lead to a significant overestimate of thickness. The observed mobility reduction and significant surface scattering in ultrathin layers are a direct consequence of stronger interlayer interaction. Carriers in BP are highly delocalized perpendicular to the plane direction, which contrasts graphene and many transition metal dichalcogenides where layer interaction is governed strictly by weak van der Waal's interaction^25^,^26^,^27^. The axis perpendicular to the basal plane in BP has a well-defined band structure and experimentally verified carrier mobility that is larger than the in-plane puckered direction^1^. Thus, surface imperfections are a more significant contribution to carrier scattering than in other multilayered van der Waal's materials. The extrapolated bulk value here is also larger than experimental estimates from previous experiments in thicker films^10^, but is similar to recent theoretical estimates^28^, cryogenic measurements on hBN encapsulated samples^12^,^29^ and bulk measurements^1^.

The tapered flake was also measured at 80 K for temperature-related mobility changes. At 80 K, due to the reduced distribution of thermal energy for carriers, *I*_on_/*I*_off_ increases for all thicknesses and off-state conductivity is suppressed. Only the thickest 13 nm region shows a small increase in band-edge mobility (marked by filled square in Fig. 3d). The temperature-independent n-type mobility of the thinner areas is consistent with increased surface scattering due to non-uniform flake thickness. This is in contrast with Fig. 1, where the device was fabricated on a flake with uniform thickness and therefore has a noticeable mobility increase from 300 to 80 K. Crystalline bulk BP and other thin p-type devices^12^,^29^ characterized in the literature have also shown a large increase in carrier mobility at low temperature, although the data shown in Fig. 1 is the first evidence of localized band-edge mobility increase.

### Pd-contacted ambipolar FETs

**Figure 4:**
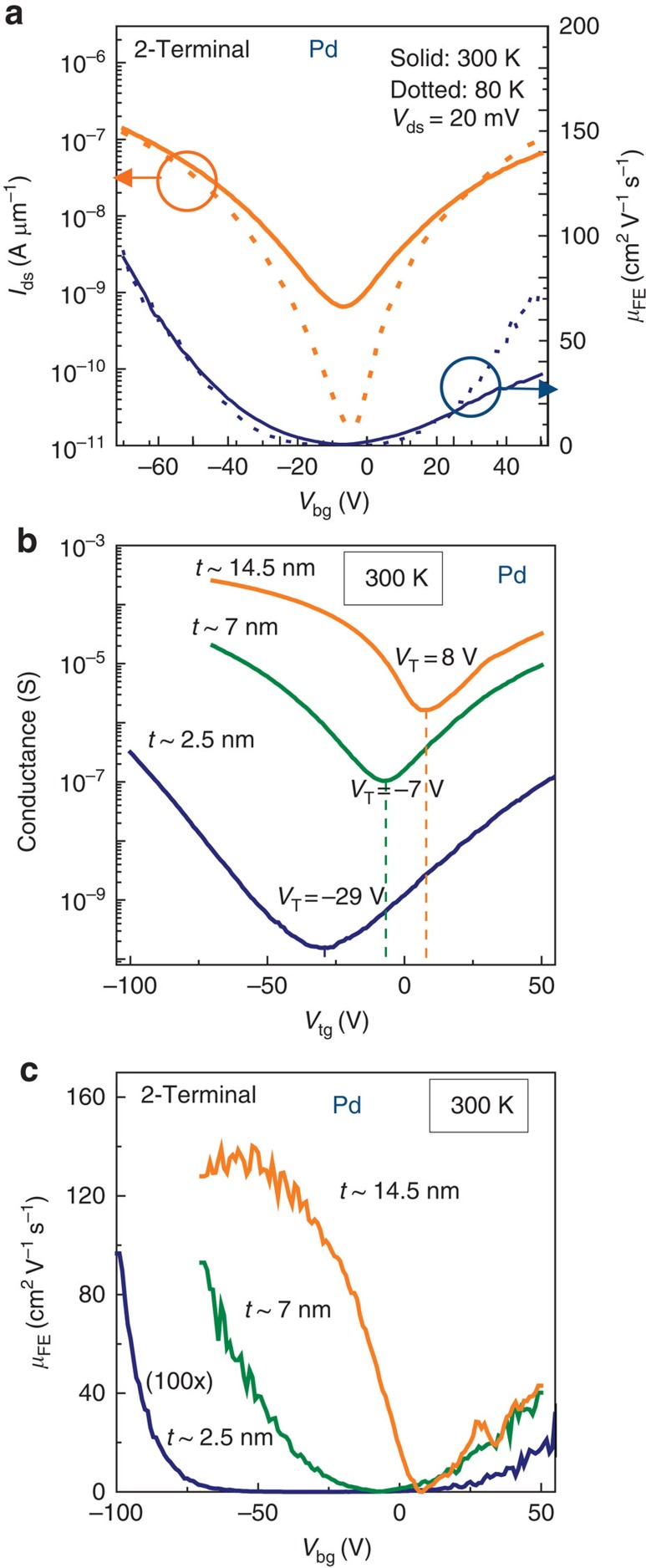
Thickness dependences of Pd-contacted BP. (**a**) Ambipolar BP-FET (7 nm thickness) measured at 300 and 80 K and the corresponding mobilities. (**b**) Thickness (*t*)-dependent *V*_T_ shift and ambipolar to unipolar conversion, consistent with decreased BP work function with increasing thickness. (**c**) Large p-type enhancement of field-effect mobility in thick flakes, while thin flakes display reduced and type-symmetric mobilities.

Before summarizing the Al Schottky-FET transport, we consider Pd, a high-work-function metal (*Φ*_m_=5.0+ eV) as a contact to BP. With Pd(10 nm)/Cr(3 nm)/Au(20 nm) contacts, ambipolar and hole-dominant operation is consistently observed for all samples in our experiments and an example is shown in Fig. 4a (see more in Supplementary Figs 4–6). Reducing temperature to 80 K improved 2-terminal electron mobility in Pd-contacted samples by 50 cm^2^ V^−1^ s^−1^, while 2-terminal hole mobility increases by only 8 cm^2^ V^−1^ s^−1^ in the measured 7-nm thick flake.

The dependence of transport on flake thickness is explored in Fig. 4b. By increasing BP flake thickness stepwise from 2.5 to 14.5 nm, the *I*_ds_–*V*_bg_ transitions from nearly symmetric ambipolar to p-type dominant, as shown in Fig. 4b,c. This coincides with a sharp positive (right) shift of the *V*_T_, and a rapid decrease of *I*_on_/*I*_off_ with increased thickness. Mobility also increases with thickness, reaching a saturated (2-terminal) 140 cm^2^ V^−1^ s^−1^ for thickness *t*=14.5 nm at 300 K. Furthermore, total suppression of electron transport is not observed for Pd-contacted FETs of any thickness, but more consistent hole-dominant transport occurs in thicker flakes.

### Summarizing thickness and contact-metal dependences

**Figure 5:**
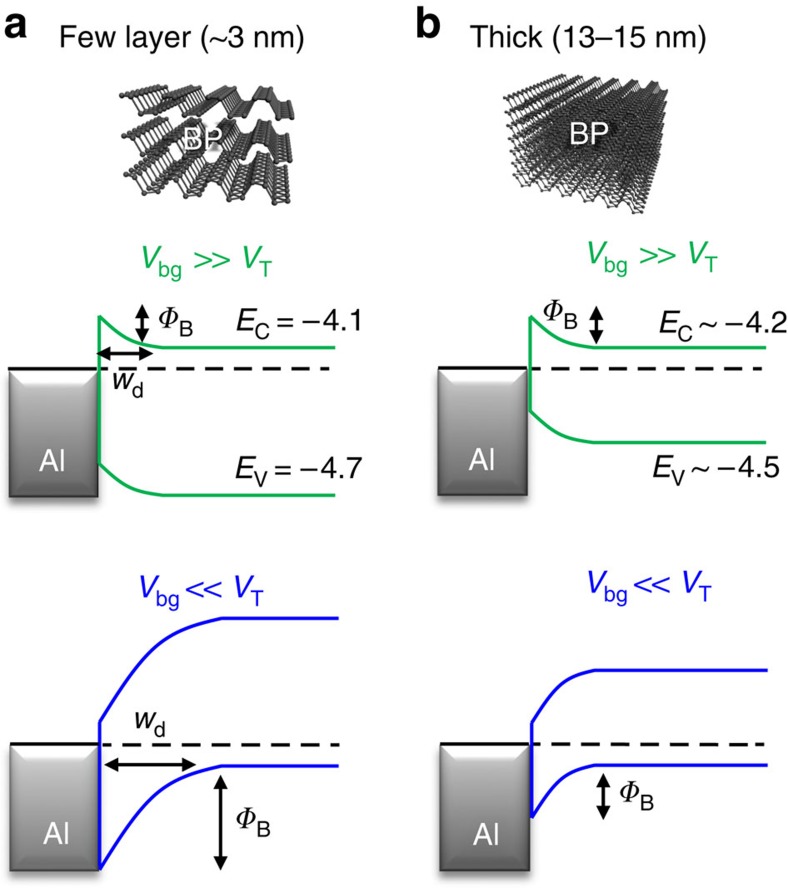
Band alignment at aluminium–BP interface. Schematic band diagrams based on previously calculated *E*_V_ and *E*_C_ (ref. 3) for (**a**) few layer (3 nm) and (**b**) thick BP flakes with Al contacts (13–15 nm). The insensitivity of *E*_C_ to thickness change and makes Al a great n-type contact but very high-resistance p-type contact with a large depletion width (*w*_d_) for thin BP. Thicker samples have small Schottky barriers for both n and p-transport, permitting ambipolar operation.

The thickness and metal dependences of BP-FETs are summarized in Table 1. Control of the dominant carrier type is a function of the Schottky barrier at the contact, akin to carbon nanotubes^30^,^31^. Aluminium works as a good n-type contact for all BP thicknesses because the conduction band minimum only weakly depends on flake thickness. Using ref. 5, 3–5 layer BP has *E*_C_∼4.1 eV and the bulk can be extrapolated to ∼4.2 eV. *E*_C_ matches well to the Al work function of 4.0–4.3 eV. For *V*_bg_>>*V*_T_ (electron-type transport), increased mobility, increased *I*_ds_ and reduced *R*_s_ with decreased temperature are evidence of a reduced Schottky barrier. Conversely, for *V*_bg_<<*V*_T_ (hole-type transport), a large Schottky barrier exists between Al and ultrathin flakes (3–3.5 nm). Since the 4-terminal measurements show this hole carrier suppression in each of 3–8 nm devices, we suggest that the Al contact depletion region extends across the extent of the 1.5–3 μm channels, holding the BP Fermi level to the conduction band minimum throughout the channel. This is further evidenced by the indiscernible n-type threshold voltage change for all thicknesses in Fig. 3d. Transport becomes more ambipolar in thicker flakes, indicating the hole-type Schottky barrier is reduced due to reduction of the bandgap. This is schematically shown in the thickness-dependent band diagrams for Al–BP in Fig. 5. Note that 2-terminal devices with Al contacts to thicker BP also have ambipolar-type transport and reduced *I*_on_/*I*_off_ (Supplementary Fig. 7). Therefore, control of the hole-type Schottky barrier is the critical factor in transport, and the unipolar n-type to ambipolar transition results from a reduction of the barrier height as flake thickness is increased.

Meanwhile, Pd contacts have previously been shown to reduce hole-type contact resistance compared with other metals^32^. Our results indicate that contact resistance of Pd to ultrathin BP (2.5 nm) is an order of magnitude smaller than that of the channel resistance (Supplementary Fig. 4). We hypothesize that the low contact resistance is related to Pd_*x*_P_*y*_ alloy formation at the contact^33^, but more rigorous study is required. This channel resistance naturally decreases as the BP thickness increases. Therefore, as BP thickness increases, the Pd–BP contact resistance will become more significant relative to the channel resistance. This is reflected experimentally in Fig. 4, where *I*_ds_ increases, and a transition from the intrinsic ambipolar to contact-influenced p-type is observed. This factor also likely contributes to the temperature independence of mobility for the ambipolar 2-terminal measurements in Fig. 4a, contrasting 4-terminal results on previous p-type samples^12^,^29^. Although previous work also demonstrated a mobility increase with 2-terminal measurements and Pd as a contact, the results are slightly different. One obvious difference is that our sample fabrication was done in a glove box, whereas in the previous approach exfoliation was done in air and shows more dominant p-type characteristics. The resulting ambipolarity of our Pd-contacted samples results in almost equal resistance for both electrons and holes, which also contrasts the p-type dominant phenomena in previous work^32^. While trivial at 300 K, at 80 K a parasitic contact resistance can mask the expected increase in BP carrier mobility. The presence of symmetric ambipolar operation in different devices is a clear sign of the quality of the samples. The p-type dominance results from the built-in potential at the contacts proportional to *Φ*_M_–*E*_V_. This induces a large band bending, whereby excess hole carriers are donated from the Pd to the BP channel. This hole doping is also found in some ultrathin films (Supplementary Fig. 6), but is mostly dominant in thicker flakes. As the thickness of BP increases, a threshold shift is observed in Fig. 4b, which is attributed to the change of *E*_V_ from −4.85 eV in trilayer to an estimated −4.5 in bulk^3^,^5^.

### Dual gating and directional transport

**Figure 6:**
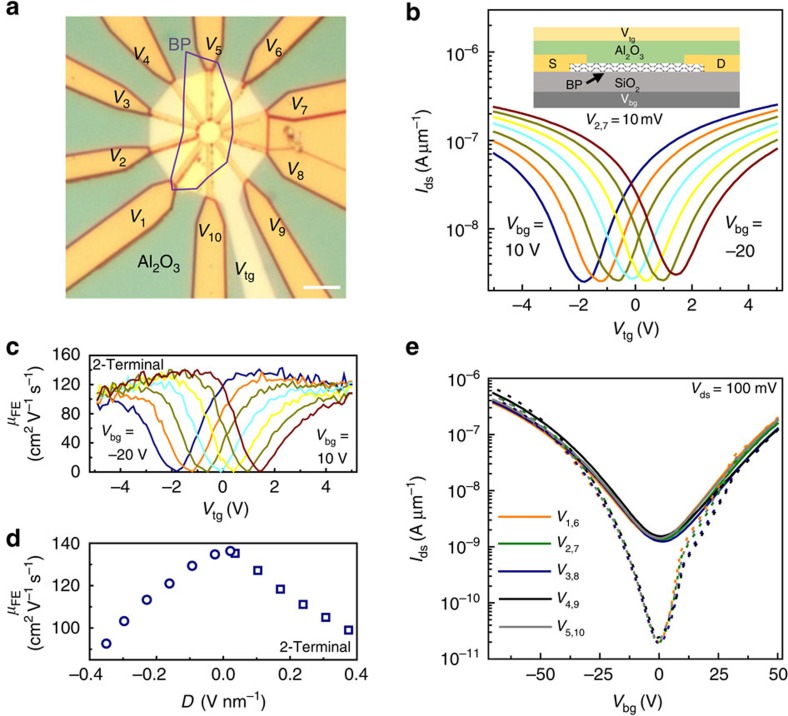
Ambipolar dual-gated BP-FETs. (**a**) Optical image of 7-nm thick dual-gated (top and bottom) device. (**b**) Fully symmetric dual-gated FET operation. The applied displacement field has no influence on device polarity. Scale bar, 5 μm. (**c**) 2-Terminal mobilities measured via the *V*_tg_ sweep. Mobility saturation is observed and electron–hole transport symmetry is retained, although peak values increased by 45 cm^2^ V^−1^ s^−1^ compared with pre-Al_2_O_3_ measurement in Fig. 4c. Inset shows the device schematic from **a**. Al_2_O_3_ dielectric thickness is 50 nm and SiO_2_ thickness is 300 nm. (**d**) Peak mobility for electrons (squares) and hole (circles) branches of the curves in **c**, plotted as a function of *D*, normalized to a thickness of 7 nm. (**e**) No clear directional dependences are observed for *I*_ds_ at both 300 K (solid) and 80 K (dotted).

To compare with the above contact metal-based control of the device polarity, we investigate the effects of dual gating and deposition of Al_2_O_3_ dielectric on channel-dominant Pd-contacted samples. In particular, we examine the influence of a vertical displacement field *D*=(*C*_t_*V*_tg_−*C*_b_*V*_bg_)/2 and surface passivation on majority carrier type and mobilities. An optical image of the sample investigated is shown in Fig. 6a. Using the top gate potential to vary the Fermi level, it can be seen that the presence of Al_2_O_3_ and induced displacement field yields no type conversion or polarity changes. Instead, symmetric hole and electron ambipolarity is retained in Fig. 6b, as in the 7 nm back-gated results in Fig. 4, which is the same device before Al_2_O_3_ deposition. However, due to all-around gate coupling and presence of a high-*κ* dielectric, a 45 cm^2^ V^−1^ s^−1^ increase in peak hole mobility from reduced surface scattering^34^, and mobility saturation are observed in Fig. 6c. Peak electron and hole carrier mobility symmetry (140 cm^2^ V^−1^ s^−1^) is also retained for all applied displacement fields, as shown in Fig. 6d. A displacement field induces a carrier concentration gradient across the BP, which is effectively a change in the channel thickness contributing to the conduction, similar to the width modulation of the strongly inverted region of a traditional buried metal-oxide-semiconductor FET. With all-around gating of BP and *D*=0, peak field-effect mobility is maximized, as shown in Fig. 6d. This bodes well for future devices; because back-gated Al-contacted BP results can be further improved. In particular, thicker flakes will benefit most since all-around gating is more effective in inverting the carrier type for more layers in the channel. By stepping *V*_bg_, the vertical displacement field across the BP varies, producing a nearly linear shift of *V*_T_ without a dominant polarity conversion or change in off-state current. This result indicates that an electric field generated either via a top gate, surface dielectric or surface functionalization (doping) is not a promising way to control a carrier type in flakes as thin as 7 nm. The influence of surface functionalization, dielectric growth or a displacement field on thinner BP flakes remains an open question.

Lastly, to ensure that the comparisons between flakes and metal contact types are not an artifact of device flake alignment, we examine directional dependences of carrier transport in Pd-contacted devices. This angle dependence is examined before and after Al_2_O_3_ gate dielectric growth. As shown in Fig. 6e, no angle dependence on carrier type is observed at 300 and 80 K in the *I*_ds_–*V*_bg_ sweeps. The angle dependence as a function of the top gate is found in Supplementary Fig. 5. This hints to anisotropic transport, but we are unable to quantify whether the small differences are related to angle. Absence of a clear directional influence is in good contrast with theoretical prediction or previous experimental report of anisotropic transport^1^,^11^,^28^. We presume that transport in the current samples is therefore dominated by extrinsic factors, not the intrinsic effective mass anisotropy present in BP. Similarly, the thickness dependences of mobility in any of the results of this communication are also not inadvertently directionally related. The quality of the samples fabricated here is evidenced by reduced hysteresis (Supplementary Fig. 6) when compared with the early reports in the literature. Improved sample quality is due to the moisture-free glove box fabrication and/or coverage by a top gate dielectric^18^,^20^,^35^. This procedure induces less residual coulomb scattering centres and trap sites in the surrounding dielectrics.

## Discussion

We have demonstrated BP transistors with unipolar n-type, ambipolar and unipolar p-type characteristics by varying contact-metal and flake thickness dependences. With Al contacts, we demonstrated the first high-performance unipolar n-type BP transistors with electron mobilities ranging from 10^2^ to 10^3^ cm^2^ V^−1^ s^−1^ and *I*_on_/*I*_off_>10^5^ at room temperature. A flake thickness dependence of dominant carrier type was also observed, which is due to the existence of a Schottky barrier at the Al–BP contact.

The excellent switching characteristics and possibility of symmetric electron and hole mobilities is promising for complementary metal-oxide-semiconductor (CMOS) integration, wherein mobility is often a limiting factor in switching speed. While the current samples are still limited by extrinsic factors such as surface roughness and contact resistance, we have demonstrated that dual gating can improve performance. The current weakness of BP is the environmental instability and lack of a scalable CVD or epitaxial growth method. If progress on these issues is successful, the ability to vary transistor operation from n-type, ambipolar or p-type via modulation of the Fermi level (electrostatic gating), Schottky barrier (contact metal, channel length and electrostatic gating) and bandgap (flake thickness) makes BP a very dynamic and useful material for logic circuits.

## Methods

### Sample preparation

BP flakes (99.998%) were purchased from smart-elements.com and stored in an inert atmosphere. Exfoliation and subsequent fabrication procedures where BP was unprotected (for example, uncovered with PMMA or Al_2_O_3_) were performed in a glove box with H_2_O and O_2_ concentration <1 p.p.m. After exfoliating flakes onto an Ar-annealed Si/SiO_2_ (300 nm) wafer and finding suitable clean locations with an optical microscope, electrodes were patterned by electron beam lithography and either Al/Cr/Au or Pd/Cr/Au was evaporated for contact electrodes. Total fabrication was completed in <24 h in all cases. Until high vacuum condition was achieved, all procedures were performed with limited or no light exposure, including storage of the flakes. Al_2_O_3_ was grown using Al_2_O_3_ dielectric with chamber temperature of 120 °C. The chamber was thoroughly cooled before growth to prevent any initial oxidation of the BP during transfer from inert environment. The oxide thickness of 50 nm corresponds to 500 cycles with trimethyl aluminium precursor.

### Field-effect mobilities

Measurements were performed in a vacuum probe station in low 10^−6^ torr vacuum with a LN_2_ feed through for 80 K measurements. The typical planar capacitance model was utilized, *μ*_FE_=*Wg*_m_/*LV*_ds_*C*_ox_, where *V*_ds_ is the applied voltage for 2-terminal and the measured probe voltages for the 4-terminal case. The *C*_b_=11.6 nF cm^−2^ for the 300-nm thick SiO_2_ back gate oxide and *C*_t_=100 nF cm^−2^ for the 50-nm thick Al_2_O_3_ top gate dielectric. Supplementary Figure 5d contains further details regarding *C*_b_ and *C*_t_. Geometry for the 4-terminal mobility calculations was defined by the central BP region between the electrodes. Details, example region and possible estimation errors are outlined in Supplementary Fig. 3.

### Schottky barrier measurement

Temperature-dependent *I*_ds_–*V*_ds_ measurements were performed while also stepping the back-gate potential *V*_bg_. *V*_ds_-dependent *Φ*_B_ was calculated from fitting the slope of the Richardson plot ln(*I*_ds_/*T*^2^) versus 1/*kT*. Equilibrium Schottky barrier height, *Φ*_B_(*V*_ds_)=0, was then extracted by plotting *Φ*_B_(*V*_ds_) versus 
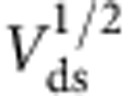
 and extrapolating the *V*_ds_=0 intercept.

## Additional information

**How to cite this article:** Perello, D. J. *et al*. High-performance n-type black phosphorus transistors with type control via thickness and contact-metal engineering. *Nat. Commun.* 6:7809 doi: 10.1038/ncomms8809 (2015).

## Supplementary Material

Supplementary Information Supplementary figures 1-7

## Figures and Tables

**Table 1:** Type control summary by thickness and contact metal.

	**BP thickness**		
	**2.5–5.5 nm**	**7–8 nm**	**13–14.5 nm**
Al contacts	Unipolar n-type	Unipolar n-type	Ambipolar
Pd contacts	Ambipolar	Ambipolar p-type dominant	Unipolar n-type
