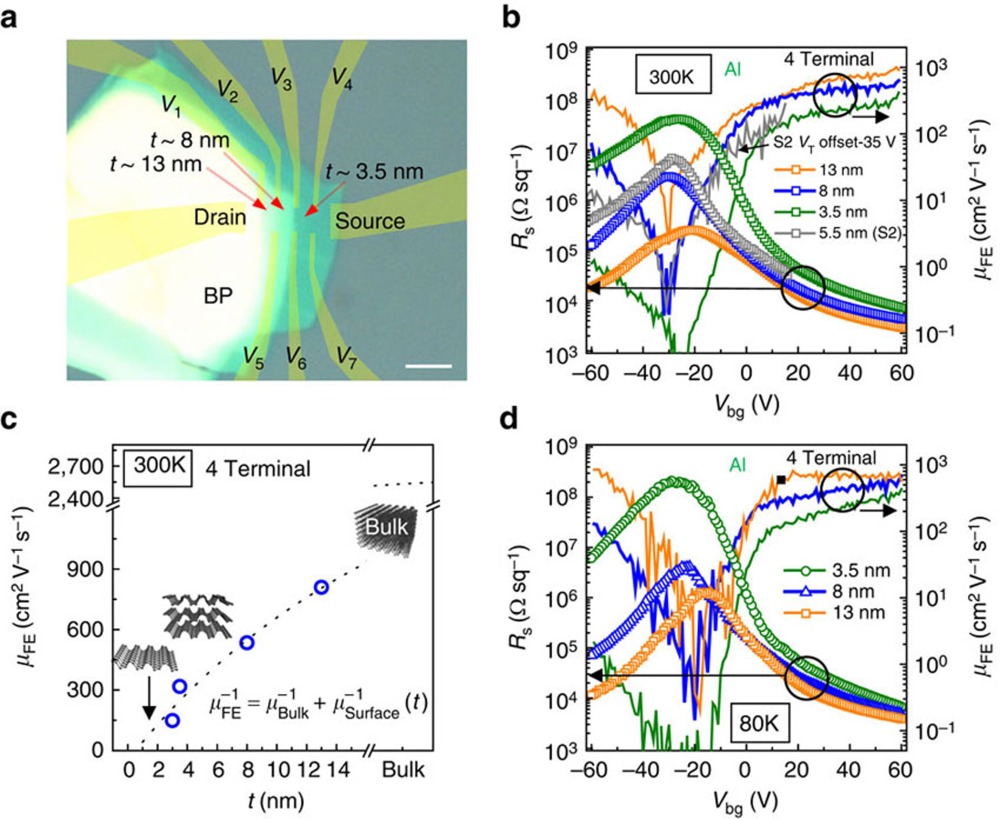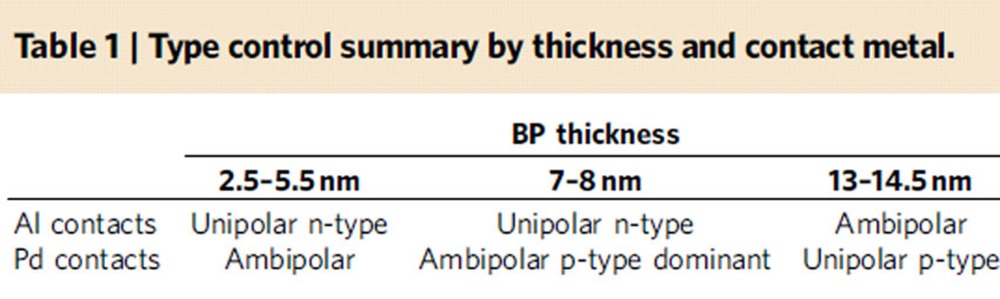# Corrigendum: High-performance n-type black phosphorus transistors with type control via thickness and contact-metal engineering

**DOI:** 10.1038/ncomms10428

**Published:** 2016-01-28

**Authors:** David J. Perello, Sang Hoon Chae, Seunghyun Song, Young Hee Lee

Nature Communications **6**, Article number: 780910.1038/ncomms8809 (2015); Published 07302015; Updated 01282016

In Fig. 3 of this article, there are a number of errors in the colours used for the data points and curves. In Fig. 3b, the blue data should be green, referring to a thickness of ‘3.5 nm', and the green data should be blue, referring to a thickness of ‘8 nm'. In Fig. 3d, the blue data should be green and refer to a thickness of ‘3.5 nm', the green data should be blue and refer to a thickness of ‘8 nm' and the orange data should refer to a thickness of ‘13 nm'.

In Table 1, the Pd contacts on 13–14.5 nm of BP were ‘Unipolar p-type', not ‘Unipolar n-type'.

The correct version of Fig. 3 and Table 1 appear below.