# Supplementary material for: High-performance n-type black phosphorus transistors with type control via thickness and contact-metal engineering
Source: Nat Commun. 2015 Jul 30;6:7809. doi: 10.1038/ncomms8809 (PMC4532797; doi:10.1038/ncomms8809)
Supplement: Supplementary Information — Supplementary figures 1-7 [file ncomms8809-s1.pdf]

Supplementary Figures

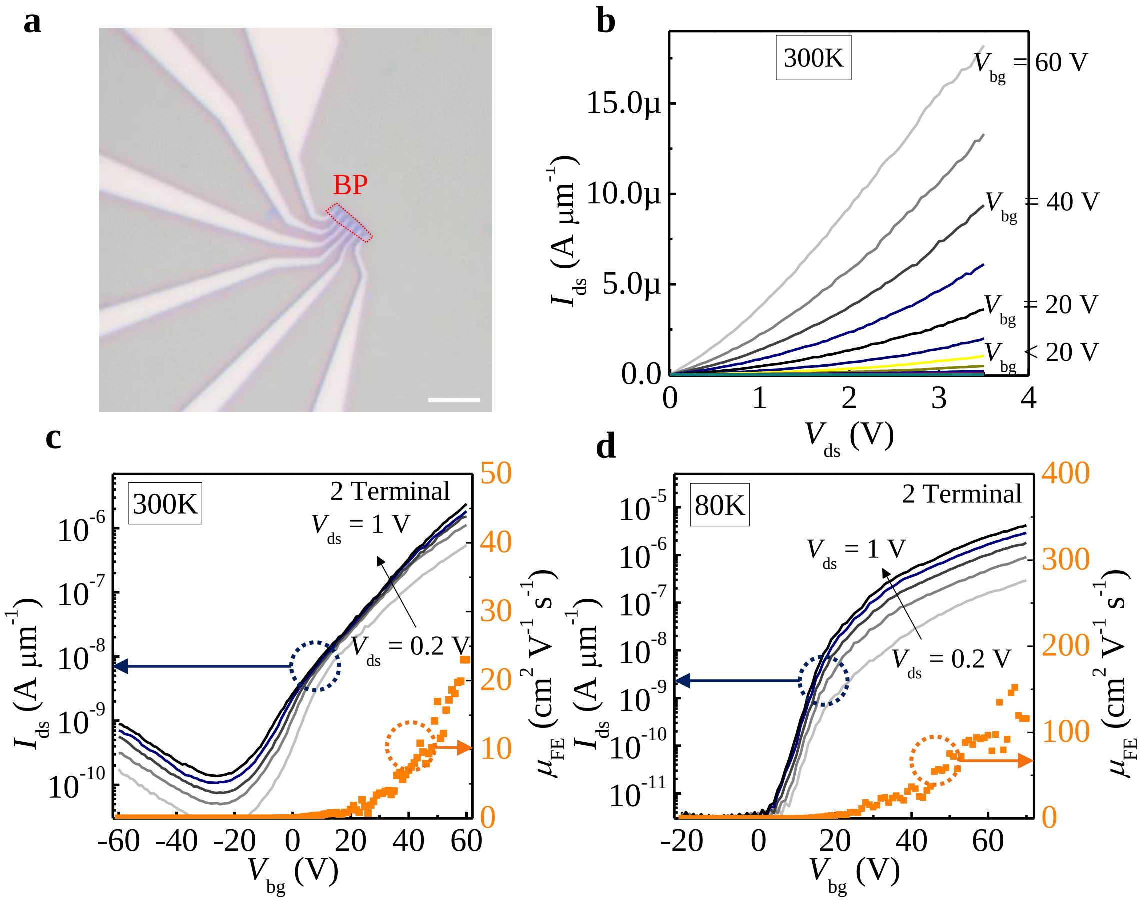

Supplementary Figure 1 | Unipolar n-type phospherene FETs. (**a**) Optical image of a sample with a series of AlCrAu electrodes fabricated onto a small ultrathin flake (3 nm). Scale bar is 3 µm. **(b-d)** One representative device of the 5 FETs on the sample in panel **a**. **(b)** I_{ds}-V_{ds} curves at 300K with n-type behavior. **(c)** I_{ds}-V_{bg} unipolar n-type behavior. 2-terminal μ_{FE} for devices in panel **a** range from 14-41 cm^2 V^{-1} s^{-1} at V_{bg} = 60 V (300K). **(d)** I_{ds}-V_{bg} at 80K with suppressed I_{off} and a 6-fold increase in μ_{FE} when decreasing temperature from 300K to 80K.

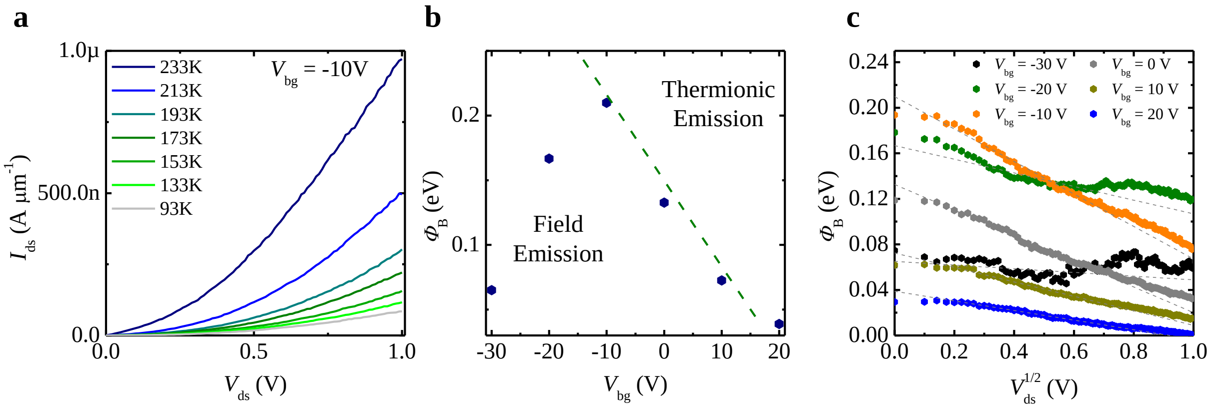

Supplementary Figure 2 | Activation energy measurements to characterize Schottky barrier (Φ_B) at the Al-BP interface. (**a**) Temperature dependence of 2-Terminal I_{ds}-V_{bg} for BP FET with flake thickness 6 nm for the device in the off-state. **(b**) Φ_B as a function of V_{bg}. (c) Φ_B-$V_{ds}^{1/2}$. The good linear fit to data for -10 V < V_{bg} < 20 V indicates thermionic emission accurately models the contact barrier. For V_{bg} < -10 V and V_{bg} > 20 V, V_{sd} has reduced or no influence on the barrier height, or a negative calculated barrier height is observed, indicating that field emission is more dominant and the thermionic emission model fails.

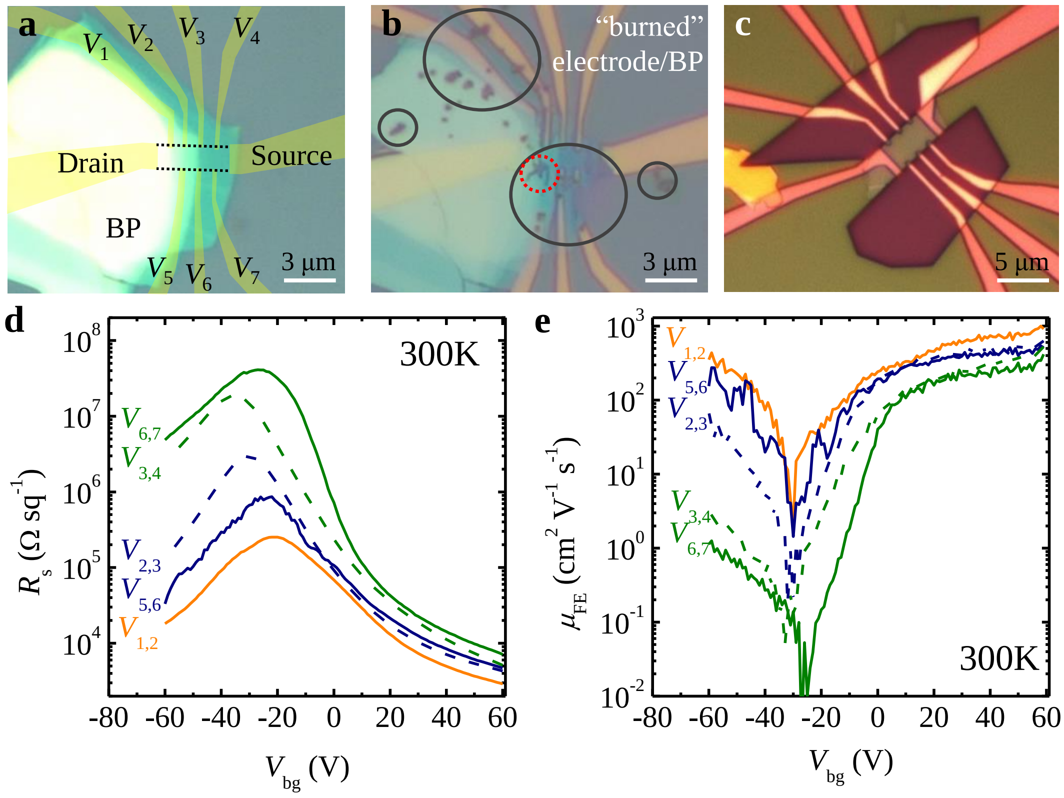

Supplementary Figure 3 | Optical Images of the tapered flake examined in the main text. (**a**) Optical image with electrodes drawn to show positions. Dotted lines mark the geometry utilized for calculating the μ_{FE} and R_s in the main text. **(b)** Optical image after measurement. Due to air exposure concerns, optical imaging is not possible between fabrication and electrical measurement. During 2-probe measurement, catastrophic device failure occurred, likely due to filament formation, e.g. leakage current, through the oxide in the position circled in red (dotted). As can be seen, the electrodes are burned and seem to "explode" with pieces landing 20-30 μm from original location. However, most of the original phospherene flake remains. It is unclear if this is due to the large current density possible in the phospherene and large amount of heat generated at the contacts. It was found that most devices that failed during measurement suffered a similar fate. **(c)** Optical micrograph of "S2" sample from which the 5.5 nm data point in the main text Fig. 3b was extracted. The BP flake was etched via RIE using 20 sccm SF_6 at 20W for 10 seconds. **(d-e)** μ_{FE} and R_s were calculated assuming the current flow dominantly occurs inside of the dotted-region in panel, e.g. assuming no current spreading due to the large Schottky barriers at the contacts. To demonstrate that this assumption is reasonable, the main text shows a comparable device with 5.5 nm flake thickness (optical image in panel **c**) which fits the trend

observed on the flake in panels **a**, **b**. Similarly, μ_{FE} and R_s are compared for opposing probe arm measurements (such as $V_{2,3}$ vs. $V_{5,6}$). As can be seen both panel **c**, **d**, the results for the on-state are consistent for both μ_{FE} and R_s. μ_{FE} and R_s for $V_{2,3}$ and $V_{5,6}$ are ~ 5% different, while $V_{3,4}$ and $V_{6,7}$ are about 15% different. Furthermore, R_s for the off-state observed for $V_{6,7}$ is slightly larger than $V_{3,4}$, although the contact area of the probe $V_{6,7}$ is nearly 3 times larger than $V_{3,4}$. This indicates that any differences are most likely just sample thickness inhomogeneity. Exceedingly precise measurement of mobilities are also not critical to the conclusions in this manuscript, and the results for either set of opposing probe arms fit the trends established in the main text.

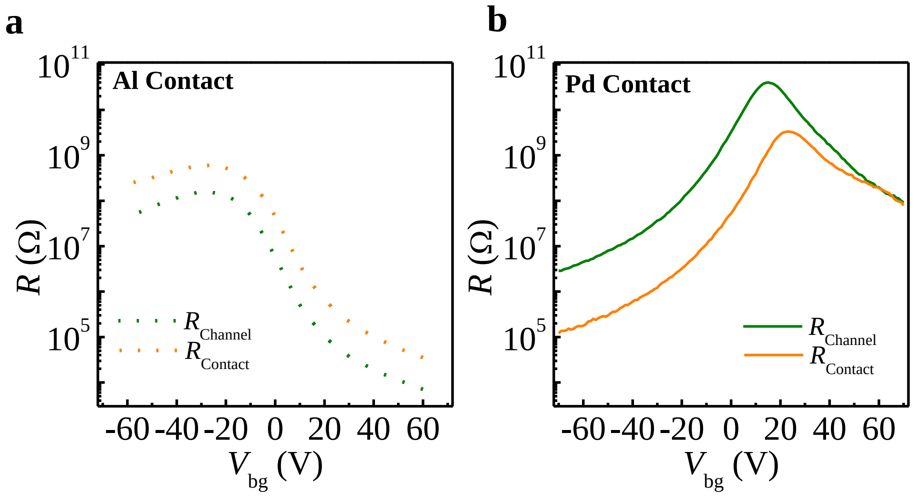

Supplementary Figure 4 | Comparing contact resistance ($R_{Contact}$) and channel resistance ($R_{Channel}$) in Pd and Al-contacted BP FETs. (a,b) Hall bar structures were fabricated on phospherene flakes. BP thickness for both flakes was 3 nm. Contact resistance was extracted by $R_{Contact} = 0.5R_{Total} - 0.5R_s(L/W)$ as a function of the back gate potential. **(a)** In Al-contacted phospherene FETs, $R_{Contact}$ is an order of magnitude greater than $R_{Channel}$, therefore Al-contacted devices act as Schottky FETs. **(b)** In Pd-contacted BP transistors, the $R_{Contact} \leq R_{Channel}$ for all V_{bg}, therefore the transistor acts as a standard MOSFET, and the intrinsic properties of the BP can be accessed.

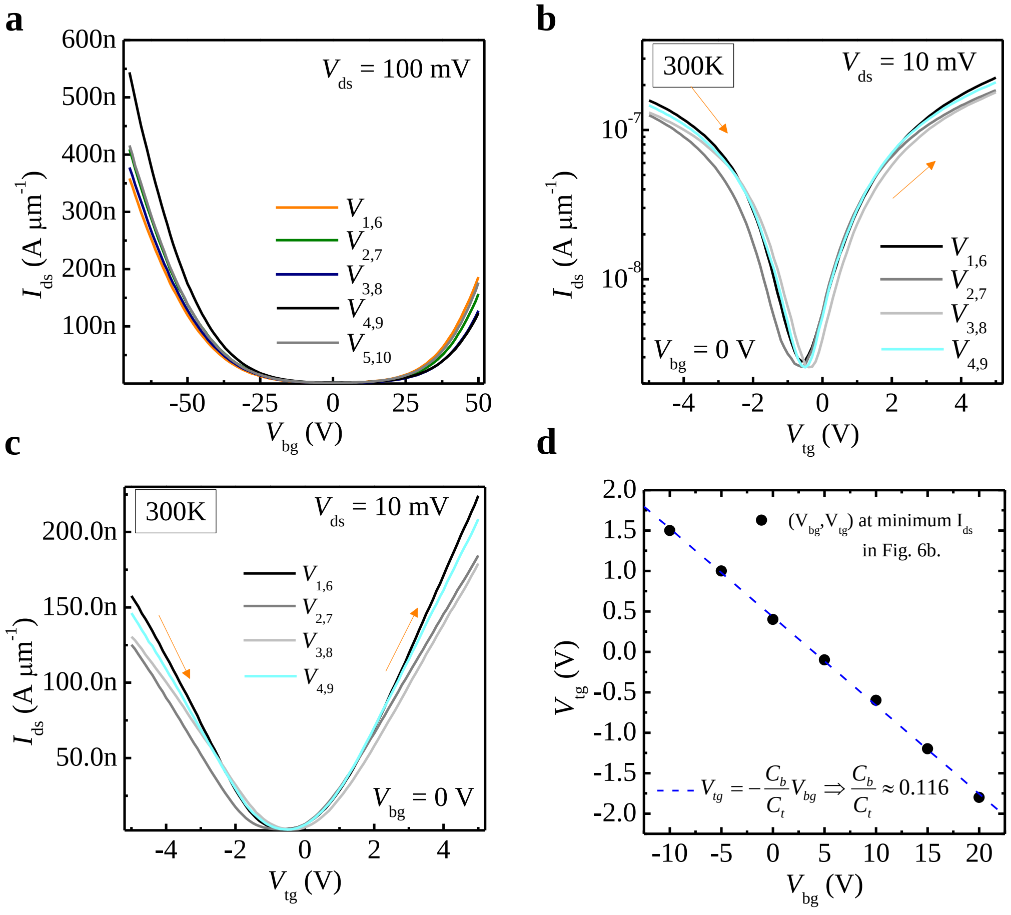

Supplementary Figure 5 | Symmetric ambipolar Pd-contacted phospherene FETs. (a) Linear scale I_{ds}-V_{bg} for different probes from Fig. 6a in the main text. **(b)** log scale and **(c)** linear scale comparison of I_{ds}-V_{tg} characteristics as a function of angle (See optical image in Fig. 6a). Nearly symmetric ambipolar response at 300K. Although differences exist in the curves, there is no clearly defined angular dependence. **(d)** Plot of V_{tg},V_{bg} at minimum conductance from each curve in Fig. 6b, which follows the relationship $V_{tg} \propto C_t^{-1} C_b V_{bg}$. Linear fitting indicates that $C_b C_t^{-1}$ ~ -0.115, giving a top gate Al_2O_3 capacitance of C_t = 100 μF cm^{-2} based on the 300 nm SiO_2 capacitance C_b = 11.6 μF cm^{-2}.

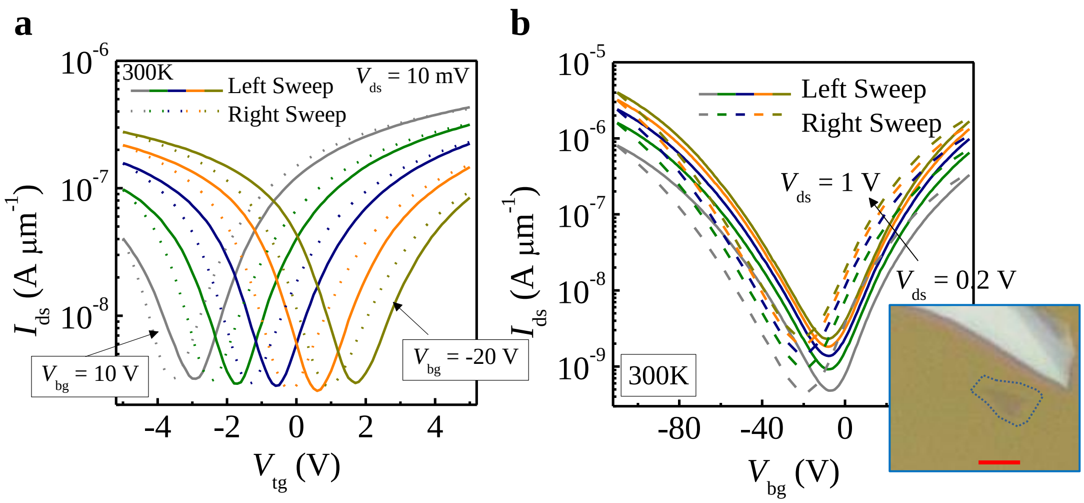

Supplementary Figure 6 | Hysteresis in symmetric ambipolar Pd-contacted phospherene FETs. (a) Hysteresis in the 2-terminal gate sweeps is about 750 mV for the I_{ds}-V_{tg} sweep for a 7 nm thick flake. The shift does not influence transport properties or symmetry considerations. **(b)** Larger 11 V hysteresis present in the I_{ds}-V_{bg} sweep for 2.5 nm flake due to the large 170 V scan range. Inset: Optical image of the measured flake prior to fabrication. Scale bar is 3 μm.

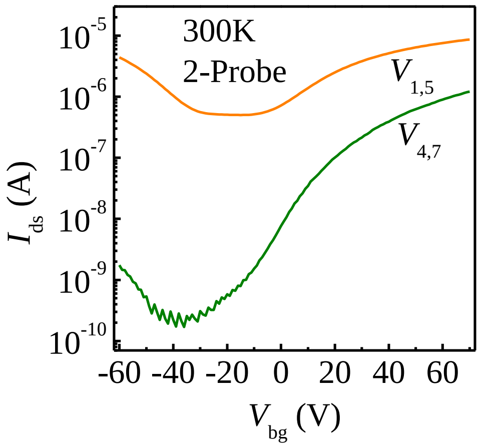

Supplementary Figure 7 | 2-terminal electrical measurements for different phospherene thicknesses. 2-terminal measurements for different phospherene thicknesses. $V_{1,5}$ shows ambipolar transport with I_{on}/I_{off} ~ 20 across 13 nm region of phospherene in Fig. 2. $V_{4,7}$ displays unipolar transport with I_{on}/I_{off} ~ 10^4 across 3.5 nm region. Clear unipolar n-type to ambipolar transition observed. Geometry is poorly defined for the two probe measurement, therefore I_{ds} is not normalized to device width.
